# BALDR: a computational pipeline for paired heavy and light chain immunoglobulin reconstruction in single-cell RNA-seq data

**DOI:** 10.1186/s13073-018-0528-3

**Published:** 2018-03-20

**Authors:** Amit A. Upadhyay, Robert C. Kauffman, Amber N. Wolabaugh, Alice Cho, Nirav B. Patel, Samantha M. Reiss, Colin Havenar-Daughton, Reem A. Dawoud, Gregory K. Tharp, Iñaki Sanz, Bali Pulendran, Shane Crotty, F. Eun-Hyung Lee, Jens Wrammert, Steven E. Bosinger

**Affiliations:** 1 Division of Microbiology and Immunology, Yerkes National Primate Research Center, Atlanta, GA USA; 2 Department of Pediatrics, School of Medicine, Emory University, Atlanta, GA USA; 3 Yerkes NHP Genomics Core Laboratory, Yerkes National Primate Research Center, 954 Gatewood Rd, Atlanta, GA 30329 USA; 4 Division of Vaccine Discovery, La Jolla Institute for Allergy and Immunology, La Jolla, CA USA; 5 Scripps Center for HIV/AIDS Vaccine Immunology and Immunogen Discovery (CHAVI-ID), La Jolla, CA USA; 6 Division of Rheumatology, School of Medicine, Emory University, Atlanta, GA USA; 7 Institute for Immunity, Transplantation and Infection, Stanford University School of Medicine, Stanford, CA USA; 8 Department of Pathology, Stanford University School of Medicine, Stanford, CA USA; 9 Department of Microbiology and Immunology, Stanford University School of Medicine, Stanford, CA USA; 10 Division of Infectious Diseases, Department of Medicine, University of California, San Diego, La Jolla, CA USA; 11 Divisions of Pulmonary, Allergy and Critical Care Medicine, Emory University, Atlanta, GA USA; 12 Department of Pathology & Laboratory Medicine, School of Medicine, Emory University, Atlanta, GA USA

## Abstract

B cells play a critical role in the immune response by producing antibodies, which display remarkable diversity. Here we describe a bioinformatic pipeline, *BALDR* (**B**CR **A**ssignment of **L**ineage using ***D****e novo* **R**econstruction) that accurately reconstructs the paired heavy and light chain immunoglobulin gene sequences from Illumina single-cell RNA-seq data. BALDR was accurate for clonotype identification in human and rhesus macaque influenza vaccine and simian immunodeficiency virus vaccine induced vaccine-induced plasmablasts and naïve and antigen-specific memory B cells. BALDR enables matching of clonotype identity with single-cell transcriptional information in B cell lineages and will have broad application in the fields of vaccines, human immunodeficiency virus broadly neutralizing antibody development, and cancer.

BALDR is available at https://github.com/BosingerLab/BALDR.

**Electronic supplementary material:**

The online version of this article (10.1186/s13073-018-0528-3) contains supplementary material, which is available to authorized users.

## Background

B cells comprise a major component of the immune system, and they function primarily by secreting antibodies that bind and neutralize discrete protein moieties on pathogens. Antibodies, also referred to as immunoglobulins (Ig) or B cell antigen receptors (BCRs), are produced by the paired expression of a “heavy chain” (IgH) immunoglobulin gene and a “light chain” (IgL) immunoglobulin gene. The unique combination of heavy and light chain genes defines the immunological activity of a B cell and also its identity, also referred to as its clonotype. In order to deal with the near infinite array of pathogenic structures that may face the immune system, B cells exhibit an incredible level of clonotypic diversity, principally achieved by recombination at the DNA level of multiple gene segments, referred to as V (variable), D (diversity), and J (joining) segments for heavy chains, and V and J segments for light chains [1]. With approximately 38–46 V, 23 J, and 6 D functional gene segments for the heavy chains and 63–71 V and 9–10 J light chain gene segments in the human genome [2, 3], the number of possible clonotypic variants is estimated to be approximately 10^14^ [4]. Given the functional importance of clonotypic diversity to immune function, the ability to investigate transcriptional information at the clonotype level would provide valuable insight into the regulatory mechanisms that regulate antibody breadth, evolution of the B cell immune repertoires, and other immunological determinants of B cell immunity.

The advent of next generation sequencing (NGS) technology has spurred the development of several tools to broadly sequence antigen receptor genes in B lymphocytes [5–7]. The earliest tools used deep sequencing of the immunoglobulin heavy or light chains, by polymerase chain reaction (PCR) amplification of the variable region, followed by MiSeq-based sequencing of the resultant amplicon. While the achievable depth of these amplicon-based approaches provided remarkable resolution (10^5^–10^6^ chains in a single experiment) [8], a significant limitation of this technology for functional studies of the immune system is that it only sequences a single chain and cannot provide information on endogenous pairing of IgH/IgL genes to definitively identify a B cell clonotype. Recently, a novel, ultra high-throughput method to identify millions of paired IgH + IgL genes was developed by Georgiou, DeKosky, and colleagues [9]. This method uses an upfront capture of individual B cells into droplets, after which an elegant in-drop PCR ligation strategy creates a single DNA amplicon containing both IgH and IgL chains for *en masse* Illumina sequencing [9]. Additionally, others have developed “medium-throughput” techniques to sequence the paired IgH and IgL repertoire; each involved single-cell sorting followed by multiplex PCR amplification in individual wells [10] or emulsions [11] yielding sequences of 1000–2000 IgH/IgL pairs. The ability to generate deep sequence data of IgH + IgL pairings constitutes a significant advance over single-chain profiling; however, it does not provide functional or transcriptional information.

Medium-scale methodologies to obtain paired T cell or B cell receptor clonotypes alongside shallow transcriptional data have recently emerged. Han, Davis, and colleagues reported the sequencing of paired T cell α/β chains along with 17 immune genes using a PCR-barcoding/MiSeq strategy in experiments that obtained data for ~ 150–300 cells [12]. Similarly, Robinson and colleagues developed a methodology for barcoding of PCR-amplified paired IgH and IgL chains from single cells that can be combined with the query of a limited set of co-expressed functional genes [13–15]. The common strategy in these techniques involved single-cell sorting into 96-well plates followed by PCR-based amplification of the paired antigen-specific receptors with a multiplex set of primers for V gene sequences and a finite set of additional genes of interest.

Recently, several groups have demonstrated that it is possible to reconstruct clonotype sequences of the paired α and β chains of T cells (TCRs) from single-cell RNA-seq data. Stubbington and Teichmann developed the TraCeR pipeline, which uses *de novo* assembly after a pre-filtering step against a custom database containing *in silico* combinations for all known human V and J gene segments/alleles in the International Immunogenetics Information System (IMGT) repository [16]. Another pipeline, VDJPuzzle [17], filters in reads by mapping to TCR genes followed by Trinity-based assembly; the total reads are then mapped back to the assemblies in order to retrieve reads missed in the initial mapping step, followed by another round of assembly with Trinity [18].

In this study, we demonstrate the utility of *de novo* assembly for the reconstruction of paired IgH and IgL of the B cell antigen receptor from single-cell RNA-seq data. We also report the development of *BALDR* (**B**CR **A**ssignment of **L**ineage using ***D****e novo* **R**econstruction), an optimized bioinformatics pipeline that recovers BCR sequences from single-cell RNA-seq data. The accuracy of paired IgH + IgL gene identification using the BALDR pipeline was validated using primary human plasmablasts obtained after seasonal influenza vaccination, and it had a clonotype identification accuracy rate of 98%. We generated a validation dataset containing 255 samples with matched NGS and reverse transcription (RT)-PCR IgH/IgL Sanger sequence data [19] and determined (1) the accuracy, recovery rate, and efficiency of four different bioinformatic immunoglobulin filtering strategies and (2) optimal sequencing parameters to minimize sequencing cost and computing time while preserving accuracy. Lastly, we applied BALDR to analyze several B lymphocyte subsets from rhesus macaques receiving novel vaccine formulations and demonstrated that, even in species with relatively poor annotation of the Ig loci, our pipeline faithfully recreates paired antibody sequences.

## Methods

### Single-cell isolation of human plasmablast and B cell subsets

Plasmablasts for single-cell RNA sequencing (sc-RNA-seq) were isolated by flow cytometric sorting from 20 × 10^6^ freshly isolated peripheral blood mononuclear cells (PBMCs) 7 days after vaccination with the seasonal 2016–2017 quadrivalent Fluarix influenza vaccine (GlaxoSmithKline (GSK), Brentford, UK), as previously described [20]. Plasmablasts were defined as CD3– CD19+ CD27hi CD38hi CD20– lymphocytes; these markers have been previously validated to specifically phenotype human plasmablasts [20]. PBMCs were stained with the following titrated mAbs at the specified concentrations in a volume of 3.5 mL phosphate-buffered saline (PBS) with 2% fetal bovine serum (FBS): CD19-FITC (6:100; Cat# 340719 RRID:AB_400118; BD Biosciences, San Jose, CA, USA), CD3-PacificBlue (3:100; Cat# 558124 RRID:AB_397044, BD Biosciences), CD38-PE (3:100; Cat# 347687 RRID:AB_400341, BD Biosciences), CD20-PECy7 (1.5:100; Cat# 560735 RRID:AB_1727450, BD Biosciences), IgD-PECy7 (3:100; Cat# 561314 RRID:AB_10642457, BD Biosciences), and CD27-APC (3:100; Cat# 17–0271-82 RRID:AB_469370, Thermo Fisher Scientific). Plasmablasts were single-cell sorted into 96-well PCR plates (Bio-Rad, Waltham, MA, USA) containing 10 μL 10 mM Tris pH 8.0 hypotonic catch buffer supplemented with RNasin at 1 U/μL (Promega, Madison, WI, USA) using a FACSAria II instrument, and were frozen immediately on dry ice, as previously described [20]. In some cases, as described in the text, plasmablasts were sorted into 10 μL of RLT buffer (QIAGEN, Hilden, Germany). Sorted samples were stored at −80 °C for long-term storage. Conventional blood B cells were defined as (CD3– CD19+ CD14– CD16–) and were sorted into 10 μL QIAGEN RLT buffer using a FACSAria II, and then immediately placed on dry ice prior to storage at −80 °C. The antibodies used for B cell staining were CD3-AlexaFluora700 (Cat# 557917 RRID:AB_396938, BD Biosciences), CD14-ECD (Cat# IM2707U RRID:AB_130853, Beckman Coulter, Pasadena, CA, USA), CD16-BrilliantViolet421 (Cat# 302037 RRID:AB_10898112, BioLegend, San Diego, CA, USA), and CD19-PC5.5 (Clone: 3–119, Cat# A66328, Beckman Coulter).

### Enzyme-Linked ImmunoSpot (ELISPOT) assay

ELISPOT was performed to enumerate influenza-specific plasmablasts present in PBMC samples. We coated 96-well ELISPOT assay mixed cellulose ester filter plates (Millipore) overnight with either the 2016/2017 Fluarix quadrivalent influenza (GlaxoSmithKline) at 1:20 in PBS or polyvalent goat anti-human Ig (Jackson ImmunoResearch, West Grove, PA, USA) at 10 μg/mL in PBS. The plates were washed and blocked by incubation with R10 media (RPMI-1640 supplemented with 10% FBS, penicillin, streptomycin, and l-glutamine) at 37 °C for 2 h. Freshly isolated PBMCs were added to the plates in a dilution series starting at 5 × 10^5^ cells and incubated overnight at 37 °C in R10 media. The plates were washed with PBS, followed by PBS/0.05% Tween, and then incubated with biotinylated anti-human IgG, IgA, or IgM antibody (Invitrogen) at room temperature for 90 min. After washing, the plates were incubated with avidin D-horseradish peroxidase conjugate (Vector Laboratories) and developed using 3-amino-9-ethylcarbazole substrate (Sigma-Aldrich). Plates were scanned and analyzed using an automated ELISPOT counter (Cellular Technology Limited (CTL)).

### Single-cell isolation of rhesus macaque plasmablast and B cell subsets

Plasmablasts were obtained by single-cell sorting from a PBMC sample obtained from a rhesus macaque 4 days after vaccination with an experimental HIV vaccine as described in [21] using the flow cytometry panel described in [22]. Single antigen-specific B cells and germinal center B cells were obtained from rhesus macaques after immunization. Single peripheral blood antigen-specific memory B cells were obtained from cryopreserved PBMCs and stained with biotin-labeled antigen-specific probes, and were further defined as CD20+ and CD4–. Splenic germinal center B cells were obtained by single-cell sorting from a cryopreserved sample and were defined without an antigen-specific probe as live, CD20+ CD38– CD71+.

### Single-cell RT-PCR amplification of immunoglobulin variable domain sequences

Single-cell sorted plasmablasts in 10 μL of hypotonic catch buffer (10 mM Tris pH 8.0, 1 U/uL RNasin (Promega)) were thawed on ice. We used 1 μL of well-mixed single-cell sorted cell lysate to generate complementary DNA (cDNA) using Sensiscript cDNA synthesis reagents (QIAGEN) according to the manufacturer’s recommended reaction conditions. The remaining 9 μL of lysate was used to generate the RNA-seq library as described below. The 1 μL of cell lysate was added to 7.5 μL of reaction mixture containing water, gene-specific primers, and 0.85 μL of 10X reaction buffer. This reaction was incubated at 72 °C for 5 min, 50 °C for 1 min, and 4 °C for 30 s, and then immediately transferred to ice. Afterwards, the reaction was brought to a final volume of 10 μL by adding 1.5 μL of a reaction master mix containing deoxynucleotides (dNTPs), 2 units of Sensiscript RT, 4 units of RNasin (Promega), and 0.15 μL of 10X reaction buffer. The reaction mixtures were then incubated at 25 °C for 10 min, 37 °C for 1 h, and 95 °C for 5 min. cDNA was stored at −20 °C prior to PCR amplification. cDNA synthesis reactions were primed using a cocktail of oligonucleotides specific for the human IgG, IgA, and IgM heavy chain constant domains and the κ and λ light chain constant domains at a final concentration of 1 μM per primer. Constant domain-specific primers were the same as those used for first round PCR amplification. Ig heavy chain and light chain (κ/λ) variable domain sequences were subsequently amplified by nested PCR using chain-specific primer cocktails encompassing all variable (V) gene families and the constant domain. PCRs were performed as previously described [19] using 2 μL of cDNA template. PCR amplicons were purified using a PCR cleanup column (QIAGEN) and sequenced by Sanger sequencing (Eurofins, North Kingstown, RI, USA) as previously described [19].

The PCRs for rhesus macaque single cells were performed as previously described [22] using an amplified SMART-Seq messenger RNA (mRNA) library (1:10 diluted).

### Single-cell RNA-seq

RNA-seq analysis was conducted at the Yerkes Nonhuman Primate Genomics Core Laboratory (http://www.yerkes.emory.edu/nhp_genomics_core). Single cells were sorted by flow cytometry into 10 μL of QIAGEN RLT buffer or hypotonic catch buffer as indicated in the text. RNA was purified using RNACleanXP Solid Phase Reversible Immobilization (SPRI) beads (Beckman Coulter). The beads with bound RNA were re-suspended in Clontech buffers for mRNA amplification using 5′ template switching PCR with the Clontech SMART-Seq v4 Ultra Low Input RNA kit according to the manufacturer’s instructions. Amplified cDNA was fragmented and appended with dual-indexed barcodes using Illumina Nextera XT DNA Library Prep kits. Libraries were validated on an Agilent 4200 TapeStation, pooled, and sequenced on an Illumina HiSeq 3000. The sequencing conditions and read depth are indicated in Additional file 1: Table S1. For the VH dataset comprising human 36 CD19+ Lin– cells, the sequencing was carried out on an Illumina MiSeq. Out of the 36 B cells, 6 were sequenced using the Clontech SMART-Seq v4. The remaining 30 were sequenced with a modified protocol where instead of using the Clontech SMART-Seq v4 kit, the cDNA was synthesized using Clontech buffers and enzymes (SMARTer method), while the template switching oligos (TSOs) were ordered from Exiqon (Woburn, MA, USA) for full-length cDNA synthesis and the primers for cDNA synthesis were ordered from Integrated DNA Technologies (Skokie, IL, USA). The libraries for the human AW1 and the rhesus BL6.1 and BL6.2 datasets were sequenced on the Illumina HiSeq 3000 twice in order to obtain greater read depth. The combined sequences from both runs for each sample were pooled prior to analysis. For the VH dataset, PCR for Sanger sequencing was performed as described above using a 1:10 dilution of 1 μL of sequencing library after the SMART-Seq amplification stage, similar to methods described for single T cells [16].

### BALDR pipeline for immunoglobulin reconstruction of human BCRs

#### Assembly

Adapter sequences were removed from fastq files using Trimmomatic-0.32 [23]. After trimming, the unfiltered or filtered reads were used as input for assembly with Trinity v2.3.2 [18] without normalization except where indicated.

#### Ig transcript filtering methods

##### IG_mapped and IG_mapped+Unmapped

The reads were mapped to the human reference genome (Ensembl GRCh38 release 86 primary assembly [24]) using STAR v2.5.2b [25]. In order to avoid missing any Ig reads due to incomplete annotation, we chose to use the coordinates for the complete loci instead of individual genes. The coordinates for the Ig loci (IGH 14:105586437–106,879,844, IGK 2:88857361–90,235,368, IGL 22:22026076–22,922,913) were obtained from the National Center for Biotechnology Information (NCBI) Gene database. Reads mapping to these coordinates were extracted from the bam file using SAMtools 0.1.19 [26] and seqtk-1.2 (https://github.com/lh3/seqtk). The resultant reads that were enriched for Ig transcripts were then used for assembly with Trinity. In addition, the Unmapped reads that were obtained from STAR were combined with these IG_mapped reads for the IG_mapped+Unmapped method prior to assembly.

##### IMGT_mapped

The human V, J, and C sequences (F + ORF+in-frame P) were obtained from the IMGT database [3]. The V, J, and C sequences were combined into a single file separately for heavy and light chains. A bowtie index was created, and the reads mapping to the IMGT sequences were obtained using bowtie2–2.9 [27] (AW2) and bowtie2–2.3.0 (AW1 and VH samples) with the following parameters: -no-unal -k 1 --local.

##### Recombinome_mapped

We designed an *in silico* database containing all possible combinations of V, J, and C sequences. This “Ig recombinome” was created using a design similar to that of a previous study detailing creation of a T cell receptor recombinome [16]. A database of all possible recombined sequences from human V, J, and C alleles obtained from IMGT was constructed. Twenty N bases were added in the beginning of the sequence for alignment with the leader sequence, and the D gene was replaced with 10 N bases. The resulting database comprised 250,250 IGH (350 V, 13 J, 55 C), 11,830 IGL (91 V, 10 J, 13 C), and 4860 IGK (108 V, 9 J, 5 C). A bowtie index was created for the heavy and light chain recombined sequences separately using bowtie2. The reads mapping to the recombined Ig sequences were obtained using bowtie2–2.9 (AW2) and bowtie2–2.3.0 (AW1 and VH samples) with the parameters --no-unal -k 1 --np 0 --rdg 1,1 --rfg 1,1.

#### Post-assembly and Ig transcript model selection

After assembly of unfiltered and filtered reads (IG_mapped, IG_mapped+Unmapped, IMGT_mapped, and Recombinome_mapped), IgBLAST v1.6.1 [28] was used for annotation of reconstructed Ig chains with the IMGT V, D, J, and C sequences as germline databases, the imgt domain system, and an e-value threshold of 0.001. The top hit was used for annotation of V, D, J, and C genes. In order to select the best model, reads used for assembly were mapped back to the reconstructed Ig sequence using bowtie2–2.3.0 (-no-unal --no-hd --no-discordant --gbar 1000 --end-to-end -a). The models were ranked according to the number of reads mapped. The models that were predicted as unproductive and models that had the same V(D)J gene annotations along with the CDR3 nucleotide sequence as a higher ranking model were filtered out. The top ranking Ig model was selected from the remaining set. The analysis was run on Amazon Web Services Elastic Compute Cloud (EC2) m4.16xlarge instances (Intel Xeon E5-2676 v3, 64 cores and 256 GB RAM) by running 8 simultaneous processes with 8 threads each.

#### Processing of Sanger sequences for the validation dataset

Sanger sequences obtained from RT-PCR were manually trimmed using Seqman Pro software in the DNASTAR Lasergene package v14.0.0.86 to remove low-quality reads at the ends. The trimmed reads were annotated with IgBLAST, and productive RT-PCR sequences were selected for validation. The reconstructed Ig chains were aligned with the PCR sequences using ncbi blastn v2.6.0 [29]. Accuracy of reconstruction was determined by comparing the V(D)J gene annotations and the CDR3 nucleotide sequence.

#### Somatic hypermutation and clonality analysis

The somatic hypermutation (SHM) levels were determined by depositing the Ig sequences reconstructed using Unfiltered method to the IMGT/HighV-QUEST web server [30]. The SHM levels were also determined for PCR sequences using the IMGT/HighV-QUEST web server. The number of mutations used does not include those resulting from N diversity.

The single cells were assigned to clonal families on the basis of shared V gene, J gene, and the CDR3 length for both heavy and light chains.

### Immunoglobulin transcript reconstruction pipeline for rhesus macaque

Ig reconstruction in rhesus macaques (*Macaca mulatta*) was carried out using four approaches: (1) Unfiltered, (2) Filter-Non-IG, (3) IG_mapped, and (4) IG_mapped+Unmapped. After trimming, the unfiltered or filtered reads were used for assembly with Trinity v2.3.2 without normalization. The Trinity assemblies were run on a local PowerEdge R630 Server (Intel Xeon E5-2620 v4, 16 cores/32 threads, 196 GB RAM) by executing 4 jobs, each with 8 threads and 32 GB RAM. The MacaM v7 genome reference was used to map the rhesus Ig loci and to remove conventional protein coding genes prior to assembly [31]. Since the Ig loci are not well annotated in rhesus macaques, the V, D, J, and C sequences from Sundling et al., 2012 [32] (available in IgBLAST), Ramesh et al., 2017 [33], and the IMGT database were aligned to the MacaM genome fasta file with blastn with an e-value threshold of 1e-5. The alignment positions were used to generate a bed file, and the coordinates were merged using BEDTools v2.26.0 [34]. The coordinates used for retrieving Ig reads were chr02a:90333086–91,387,066; chr02a:108598746–108,953,331; chr05:24850435–24,889,290; chr09:31850493–31,851,761; chr14:33784130–33,784,611; chr14:168090141–169,063,206; chr14:169167858–169,720,918; chr15:58889859–58,901,394; chr15:62387209–62,387,505; chr15:63455638–64,109,298; chr15:64226628–64,285,171; chr15:64411063–64,745,369; chr15:65440882–65,445,469; chr15:66221918–66,222,233. The reads were mapped to the MacaM reference using STAR, and Ig reads were retrieved with SAMtools and seqtk as done for human samples. The Unmapped reads were obtained from STAR and merged with IG_mapped reads and then assembled. For the Filter-Non-IG method, reads that mapped to annotated genes (non-Ig) in the rhesus genome were filtered out, and the assembly was run with the remaining reads. The post-assembly analysis was similar to that for the human analysis pipeline. For annotation, we used the sequences available from IgBLAST (original source [32]).

## Results

### Experimental design

**Fig. 1:**
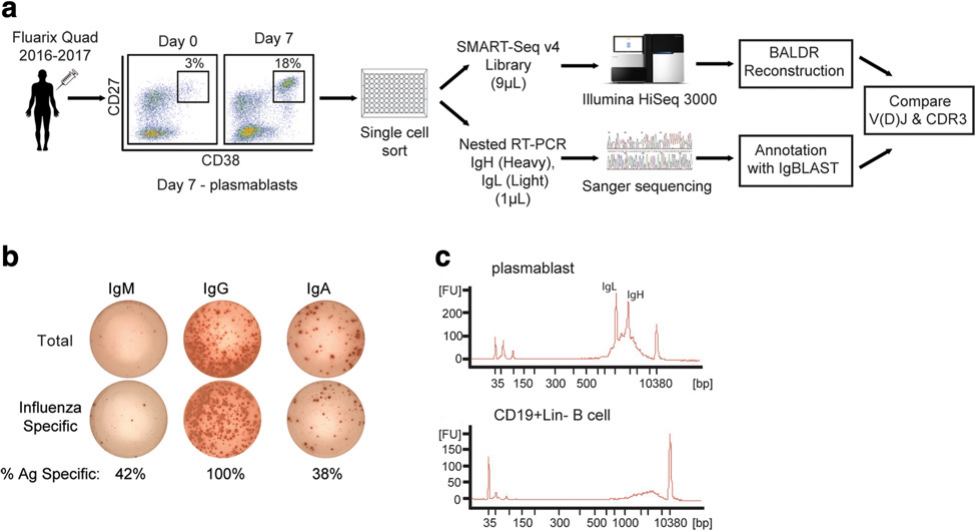
Experimental design. **a** A healthy individual was vaccinated with Fluarix Quad 2016–2017 vaccine and after 7 days CD38+ CD27+ plasmablasts were single-cell sorted into 96-well plates using flow cytometry. 10 μL lysates were aliquoted to single-cell RNA-seq (9 μL) and nested RT-PCR (nested RT-PCR (1 μL)) to sequence the immunoglobulin heavy (IgH) and light (IgL) chain genes. **b** ELISPOT assay of day 7 post-vaccination plasmablasts that shows IgH isotype usage and specificity of the plasmablast population for influenza vaccine. **c** Bioanalyzer plots of single-cell sequencing libraries after SMART-Seq v4 amplification for a plasmablast and a peripheral blood CD19+ B cell. The peaks in the plasmablast plot match in nt sequence length to the full-length heavy and light chain genes. *Ig* immunoglobulin gene, *IgH* immunoglobulin heavy chain gene, *IgL* immunoglobulin light chain gene

The goal of this study was to design and test a method for reconstructing accurate nucleotide sequences of rearranged immunoglobulin heavy and light chain genes from single-cell RNA-seq data. Plasmablasts are a class of B cell that is present at low frequencies in blood under steady-state conditions, but these cells undergo a rapid, transient expansion approximately 4–7 days after vaccination. To obtain a suitable population of plasmablasts enriched for vaccine-specific cells, plasmablasts were sorted as previously described [19] from blood collected from healthy human donors at day 7 after vaccination with the 2016/2017 Fluarix quadrivalent vaccine during the 2016 autumn flu season (Fig. 1a). Plasmablasts are a particularly useful population to query emergent B cell responses, as they are highly enriched for antigen-specific cells, and they allow for unbiased interrogation of relevant, vaccine-induced B cells without using fluorescently labeled antigenic probes or other technologies. Consistent with previous data [19, 35, 36], plasmablasts were massively expanded at 7 days post-vaccination, and were nearly 100% antigen-specific (Fig. 1b). We generated a dataset of sc-RNA-seq transcriptomes from 176 plasmablasts (Additional file 1: Table S1), obtained by flow cytometric sorting single B cells into 10 μL of lysis buffer of 96-well plates. We used 9 μL of the 10 μL cell lysate as input material into SMART-Seq mRNA amplification library preparation (Fig. 1a). After cDNA amplification of single plasmablasts, prominent peaks representing the IgH and IgL mRNA were readily apparent by microcapillary electrophoresis (Fig. 1c). The remaining 1 μL of lysate was used for conventional RT-PCR and Sanger sequencing of the heavy and light chain genes (Fig. 1a). In total, we generated a dataset of 255 Ig chains (115 heavy and 140 light chains) from Sanger sequencing with which to test the accuracy of our pipeline. Out of the 176 cells, 159 cells had at least one Ig chain represented in this dataset, while 96 cells had both the heavy and light chains (Additional file 1: Table S1).

### Pipeline to reconstruct paired immunoglobulin sequences

**Fig. 2:**
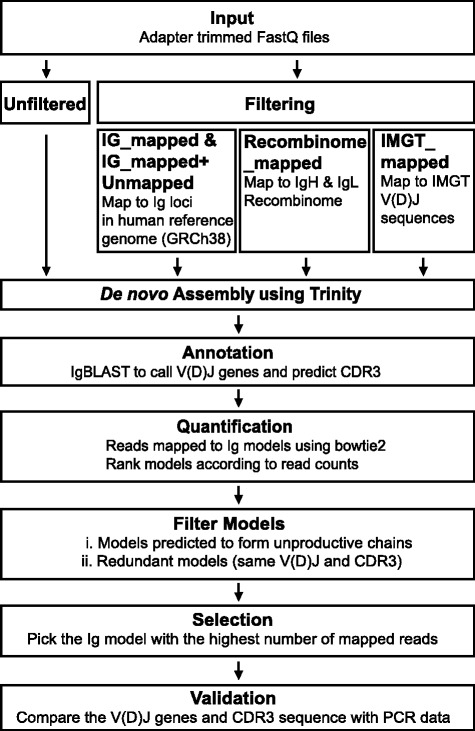
Pipeline for immunoglobulin gene reconstruction in human samples. The pipeline used for IgH and IgL gene reconstruction using either all sequencing reads (Unfiltered) or bioinformatically filtered reads (IG_mapped, IG_mapped+Unmapped, Recombinome_mapped, and IMGT_mapped) from sc-RNA-seq data. Details for each filter are described in Methods and in the text. In the initial step, adapter sequences are trimmed from the fastq files using Trimmomatic. Reads are then filtered to enrich those containing partial sequences from the IgH or IgL variable region and constant regions, and to exclude reads mapping to conventional protein coding genes. Filtered (or total) reads are then assembled using the Trinity algorithm without normalization. The assembled transcript models are annotated using IgBLAST. The reads used for assembly are mapped to the assembled transcript models using bowtie2. The models are ranked according to the number of reads mapped. Transcript models that are not productive or have a V(D)J and CDR nucleotide sequence that is the same as a higher ranked model are filtered out. The top model from the remaining set is selected as the putative heavy or light chain

An overview of the bioinformatics pipeline is shown in Fig. 2. The pipeline comprises the following major stages: (1) adapter trimming, (2) filtering of reads to enrich immunoglobulin transcripts, (3) *de novo* assembly of contiguous reads using the Trinity assembler, (4) annotation of Ig transcript models with IgBLAST, (5) read quantification, and (6) filtering of non-productive or redundant Ig transcript models. Models were then selected based on having the highest number of mapped reads, and validated with the Sanger sequencing data.

**Fig. 3:**
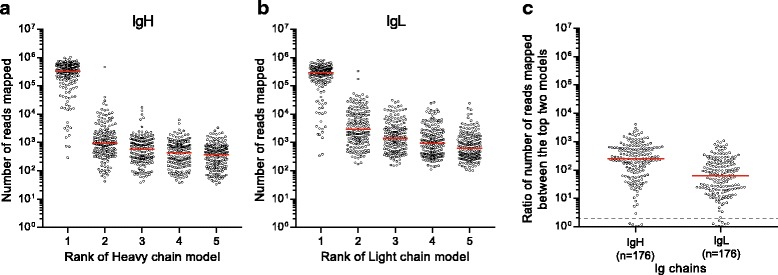
*De novo* reconstruction of sc-RNA-seq data yields a single dominant transcript model for IgH and IgL. The number of sequencing reads mapping to the reconstructed Ig transcript models (IG_mapped+Unmapped method) using bowtie2 quantification are shown for 176 flu vaccine-induced human plasmablasts (AW2-AW3 dataset). **a** IgH transcript models using Unfiltered reconstruction. **b** IgL models from Unfiltered reconstruction. **c** Ratio of reads mapping to the top and second-most abundant transcript models from Unfiltered reconstruction for IgH and IgL. The *dashed line* indicates a twofold ratio between the top and runner-up models. *Red lines* represent medians of each dataset

Adapter sequences used for library preparation were trimmed from the sequenced reads using Trimmomatic [23]. Trimmed reads were then assembled using Trinity. *De novo* assembly is a highly computationally intensive task, and scalability becomes a significant limitation in single-cell studies that involve analysis of hundreds or thousands of cells. In order to overcome this bottleneck, four different filtering strategies were evaluated for selecting Ig-specific reads. The first filtering strategy (termed IG_mapped) involved mapping of reads to the Ig loci in the human reference genome (GRCh38) using the STAR aligner [25]. Reads mapping to the three major Ig loci (IGH chr14, IGK chr2, and IGL chr22) were selected and assembled with Trinity. Due to the highly divergent nature of Ig sequences, it is possible that some reads may not map to the Ig loci in the reference genome. As a result, we also tested a filtering strategy that included unmapped reads (reads not mapping to the GRCh38 reference genome) in addition to the reads mapping to the major Ig loci (IG_mapped+Unmapped). The third filtering strategy involved creating an *in silico* “Ig recombinome” database of all possible combinations of human V, J, and C genes from IMGT, similar to a previously described strategy for T cells [16]. Sequencing reads that mapped to the recombined sequences were retained for assembly (Recombinome_mapped). Lastly, in our fourth strategy, (IMGT_mapped) reads were mapped to the IMGT database [3] of human V, D, and J sequences and extracted for assembly. We also tested assembly of all reads without filtering (Unfiltered). After running Trinity assembly to build contig models of the remaining transcripts, IgBLAST [28] was used on assembled Ig sequences for V(D)J gene annotation, prediction of the CDR3 sequence, and to determine whether the Ig chain was productive. We observed that assembly of RNA-seq reads can result in several Ig transcript models (Fig. 3). For selecting the most representative model, all reads used for assembly were mapped to each Ig model. Ig transcript models were ranked according to the number of reads mapped and then filtered to remove (1) models predicted to be unproductive and (2) models having the same V(D)J genes and the CDR3 sequence as a higher ranked model. The top ranking model that remained after filtering was then selected for validation with nested RT-PCR-derived sequences.

### *De novo* assembly of plasmablast sc-RNA-seq data yields a single dominant assembly model of IgH and IgL transcripts

As discussed above, assembly of RNA-seq reads results in multiple putative assembly models for Ig transcripts. However, we observed that each cell was found to have a dominant heavy and light chain model with all the evaluated methods, regardless of filtering approach (Fig. 3 and Additional file 1: Figure S1). The median number of reads mapping to the first and second most prevalent reconstructed heavy chain assembly models from our preferred filtering method, IG_mapped+Unmapped, was 334,090 and 937, respectively (Fig. 3a). Similarly, the median read count for the top and the second most abundant assembly models for light chains was 289,539 and 2896, respectively (Fig. 3b). The median ratio of mapped reads for the top model relative to the runner-up model was 250-fold and 61-fold for heavy and light chains, respectively (Fig. 3c). Of note, we observed that of the 176 cells, five had a ratio of the top model:runner-up of less than two-fold for IgH (Fig. 3c), and eight had ratios of less than two-fold for IgL. Collectively, these data indicate that *de novo* assembly, with or without filtering, is able to provide an unambiguous transcript model for the IgH and IgL chains in 93–98% and 95–97% of cells, respectively.

### Immunoglobulin reconstruction accuracy is near 100% at the clonotype and nt levels

**Fig. 4:**
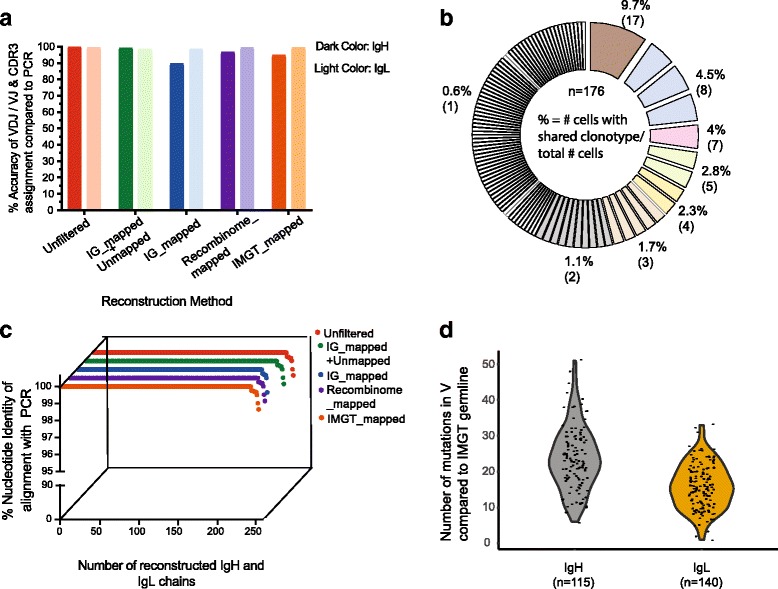
Reconstruction of Ig transcripts by BALDR is highly accurate. The fidelity of bioinformatic reconstruction of immunoglobulin variable regions was assessed by sequence comparison to a “gold-standard” sequence obtained independently from an aliquot of the single B cell lysate prior to amplification. **a** Accuracy, defined as correct identification of clonotype (V(D)J gene segment and CDR3 sequence of NGS-reconstructed IgH and IgL relative to 115 IgH and 140 IgL sequences obtained from nested RT-PCR and Sanger sequencing for all filtering methods. **b** Clonal distribution of single cells. The cells were assigned into families based on V, J, and CDR3 length of IgH and IgL. **c** Assessment of NGS-reconstruction fidelity at the nt level. Nucleotide sequences of reconstructed IgH chains determined to be accurate at the clonotype level were compared to matched sequences obtained by Sanger sequencing by blastn alignment. **d** SHMs in V region compared to germline IMGT sequences

We next assessed the accuracy of each method to reconstruct IgH and IgL chains from single-cell NGS data by comparing the reconstructed sequences to matched sequences obtained by conventional nested RT-PCR/Sanger sequencing [19]. We defined overall accuracy as the fraction of IgH and IgL chains in which reconstruction correctly called the V(D)J gene usage and CDR3 sequence relative to the RT-PCR/Sanger matched reference sequences in the 115 samples with matched NGS + PCR heavy chain sequences and 140 samples with matched light chain sequences (Fig. 4a). A high recovery of reconstruction was observed, regardless of filtering method, for IgH chains, as all methods successfully reconstructed a productive chain in all samples, with the exception of IG_mapped filtering, which had 98% recovery of IgH chains (Additional file 1: Figure S2A and Table S2). Out of the 176 plasmablasts sequenced, all filtering methods were able to yield productive IgL chains for 100% of samples (Additional file 1: Figure S2A and Table S2). Reconstructions using the Unfiltered approach showed the highest concordance (115/115 IgH (100%) and 139/140 IgL (99.3%)) with RT-PCR results (Fig. 4a, Additional file 2). Using the best filtering method (IG_mapped+Unmapped), the accuracy for IgH was 99.1% (114/115 chains) and for IgL was 99.3% (139/140 chains) (Fig. 4a). Recombinome_mapped filtering showed 111 IgH (96.5%) and 139 IgL (99.3%), and filtering against IMGT_mapped 109 IgH (94.7%) and 139 IgL (99.3%) (Fig. 4a, Additional file 1: Table S2, Additional file 2). A significant dropoff in accuracy in clonotype determination for the heavy chain was observed for the IG_mapped filtering method (103 IgH (89.5%) and 139 IgL (99.3%)) (Fig. 4a, Additional file 2). In general, the accuracy of reconstruction was higher for the less diverse light chains compared to the heavy chains. Evaluation of BALDR’s accuracy rate for yielding paired clonotype information showed that it was able to get accurate reconstructions for both IgH + IgL chains in 98.9% of the 96 cells where we had paired IgH-IgL sequences from RT-PCR with the Unfiltered method. IG_mapped+Unmapped showed the next best accuracy with accurate reconstructions in 94 out of the 96 cells (97.9%), followed by Recombinome_mapped (94.8%) and IMGT_mapped (92.7%), and again, a substantial dropoff was seen for the IG_mapped method (88.5%) (Additional file 1: Table S2). Collectively, these data demonstrate that our Ig chain reconstruction pipeline can efficiently and accurately determine the clonotype usage of plasmablasts from sc-RNA-seq data.

To assess if our accuracy estimates could be biased by clonotypes that were overrepresented in the dataset, we calculated the degree of clonality (Fig. 4b). We found that the 176 plasmablasts exhibited high clonality (Fig. 4b, Additional file 3) with the largest clonal family comprising 9.7% of the cells. We recalculated the accuracy considering the clonotype and found that the accuracy for Unfiltered method remained high at 100% for IgH, 98.8% for IgL, and 98.3% for paired IgH-IgL as well as the IG_mapped+Unmapped method (98.5% for IgH, 98.8% for IgL, and 96.6% for paired IgH-IgL) (Additional file 1: Table S3). Investigation into the reason for the loss of accuracy using the IG_mapped filtering method, which relies on retaining reads that map to the GRCh38 genome reference, revealed that for cells that had yielded incorrect IgH assembly models, these models had a substantially lower number of reads mapping when compared to the correct model yielded by the Unfiltered method (Additional file 4). In the majority of cases, we found that the “correct” V gene was incorporated into models with high read count, but these models were non-productive and filtered out (data not shown). The inclusion of unmapped reads (i.e., using the IG_mapped+Unmapped method) rescued these IgH models. This difference in accuracy between a method that relies solely on mapping to a reference (IG_mapped) compared to one that adds unmapped reads (IG_mapped+Unmapped) demonstrates the value in retaining unmapped reads, which helps to retain reads that may be otherwise lost due to incompleteness of a reference, allelic diversity or SHM.

Having determined the accuracy of clonotype assignment, we next examined the fidelity of reconstruction at the nucleotide level. The nucleotide sequences of reconstructed Ig chains were compared to the 255 RT-PCR generated sequences using blastn (Fig. 4c, Additional file 1: Figure S2B). In the vast majority of cells, the reconstructed sequences showed 100% nucleotide identity to the PCR-derived sequences (Fig. 4c). We observed that 96.5% of the reconstructed heavy and light chains had zero mismatches or gaps across all methods (Additional file 1: Figure S2). Of the remaining sequences that were not an exact match, the nucleotide identity exceeded 98.6% (Additional file 1: Figure S2). To ensure that our estimates of nucleotide identity were not biased by short alignments, we also considered the degree of sequence coverage in the reconstructed chain compared to the RT-PCR data. Out of the 255 chains, the sequence coverage was greater than 97% for 254 chains with Unfiltered and IG_mapped+Unmapped methods, 252 with Recombinome_mapped and IMGT_mapped, and 246 for IG_mapped (Additional file 1: Figure S2). Of note, we calculated the degree of SHM in the 176 plasmablasts and found it to be relatively high (median 23 nt changes from germline for IgH, 16 for IgL) (Fig. 4d, Additional file 5). Overall, these data demonstrate that our reconstruction pipeline faithfully reconstructs Ig transcript nucleotide sequences and has the ability to detect nucleotide changes induced by junctional diversity and SHM between individual cells in a clonal lineage.

*De novo* reconstruction of NGS data typically involves substantial computational resources, and a significant practical consideration of our pipeline is the computing time needed for assembly of each sample. We tested the computation times needed for each filtering method for Trinity assembly (Additional file 1: Figure S3). The median assembly time for a plasmablast cell was 2831 s (47 min) for the Unfiltered method, 310 s (5.2 min) for IG_mapped+Unmapped, 211 s (3.5 min) for IG_mapped, 317 s (5.3 min) for Recombinome_mapped, and 316 s (5.3 min) for the IMGT_mapped filtering methods. The time taken for assembly of Unfiltered reads was more than ninefold higher compared to filtering methods for enriching Ig transcripts. Taken together with the accuracy rates, these data demonstrate that Ig-transcript filtering significantly reduces the computational burden for assembly, with a negligible impact on accuracy.

The most recent version of the Trinity assembly software provides a feature for *in silico* normalization of reads to reduce the computation time for assembly. We found that running Trinity with the normalization feature resulted in reduced accuracy for Ig reconstruction in most cases (Additional file 1: Figure S4, Additional file 2). However, for the Recombinome_mapped and IMGT_mapped methods, normalization was found to slightly improve the accuracy by 2% and 3%, respectively.

### BALDR reconstructs paired Ig chains in conventional B cells

**Fig. 5:**
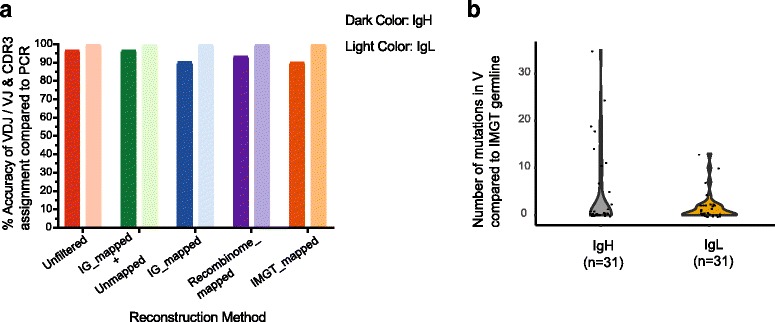
Accurate Ig reconstruction in conventional human CD19+ B cells. **a** Accuracy of Ig reconstruction for peripheral blood total CD19+ B cells (VH dataset) determined by comparison to 31 IgH and 31 IgL sequences obtained from nested RT-PCR and Sanger sequencing. **b** Somatic hypermutations in V region compared to germline IMGT sequences

Plasmablasts are a unique cell population in that approximately 5–50% of the mRNA transcriptome (Additional file 6) comprises transcripts for the immunoglobulin heavy and light chain genes. To test our pipeline on a B cell population in which the immunoglobulin transcripts were less abundant, we sorted conventional, peripheral blood B cells (defined as CD19+ CD3– CD16– CD14–) cells from a healthy donor as single cells (Additional file 1: Table S1). At least one productive sequence for each heavy and light chain was reconstructed for all 36 B cells. Due to the lower amount of Ig RNA, nested RT-PCR was carried out from the amplified SMART-Seq mRNA library, rather than from a portion of the single-cell lysate. Thirty-one IgH and 31 IgL high-quality Ig sequences were obtained from Sanger sequencing of nested RT-PCR Ig chains. Comparison of the V(D)J genes and the CDR3 sequence with the 62 RT-PCR sequences showed that Ig chains can be reconstructed accurately even in B cells with much lower levels of Ig transcripts (Fig. 5a, Additional file 2). All methods showed 100% (31/31 chains) accuracy for light chain reconstruction. The accuracy for the heavy chain ranged from 90.3% (28/31 chains) to 96.8% (30/31 chains) with Unfiltered and IG_mapped+Unmapped having the highest accuracy. A dominant heavy and light chain model was also observed in all B cells similar to plasmablasts (Additional file 1: Figure S5 and Table S4). In contrast to plasmablasts, where ~ 39% of all RNA-seq reads were Ig, the percentage of Ig reads in B cells ranged from 0.2 to 7.9% with a median of 2.2% (Additional file 6), and the majority of B cells had low or absent levels of SHM (Fig. 5b).

### BALDR maintains accuracy across a broad array of sequencing parameters

The 176 plasmablast cells described thus far were sequenced using single-ended 151-base reads (SE 151). However, sc-RNA-seq data can be generated with varying configurations of read length and/or single vs paired ends. To test the effect of these sequencing parameters, we generated a new sc-RNA-seq dataset of 101-base paired-end reads using 86 plasmablasts from another healthy individual obtained 7 days after influenza vaccination. We also generated a new matched dataset of IgH and IgL sequences from RT-PCR in which the starting material was 1 μL of unamplified lysate. We were able to get high-quality sequences for 34 IgH chains and 41 IgL chains with RT-PCR. To test the effect of sequencing parameters on clonotype assignment accuracy, we generated datasets simulating alternate sequencing parameters by truncating the 101-base reads to 75-base and 50-base reads *in silico*, and by omitting the second read of the mate pair. As above, the accuracy of the reconstructed Ig chains was determined by comparing the V(D)J gene annotation and the CDR3 sequence with the RT-PCR sequences.

**Fig. 6:**
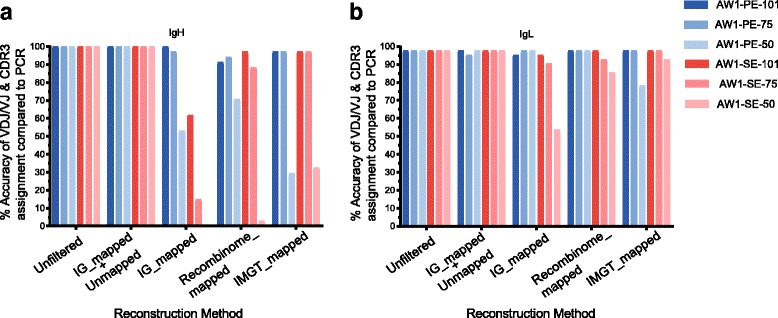
BALDR maintains accuracy across diverse sequencing parameters. Accuracy of Ig reconstruction for 51 plasmablasts (AW1 dataset) for different sequencing conditions (PE/SE and read lengths of 50, 75, and 101) determined by comparison to 34 IgH (**a**) and 41 IgL (**b**) sequences obtained from nested RT-PCR and Sanger sequencing. *PE* paired end, *SE* single-end sequencing

The Unfiltered and the IG_mapped+Unmapped methods showed the same accuracy, 100% for IgH chains and 97% for IgL chains (Fig. 6, Additional file 1: Table S5, and Additional file 2). The IgL chain did not match the reconstructed sequences for only one sequence out of 41. These methods showed the same accuracies across all the sequencing conditions tested. Comparatively, the accuracy derived from data filtered with the IG_mapped, Recombinome_mapped, and IMGT_mapped methods were much more sensitive to reductions in read length. Mapping-based approaches showed a decline in accuracy with decreasing read length, and the decline was much higher for heavy chains compared to the light chains (Fig. 6). IG_mapped and Recombinome_mapped also showed better accuracies for paired-end sequencing. For IMGT, using paired-end sequencing showed less accuracy, since concordantly mapping reads may not be obtained with the small J sequences. Collectively, these data demonstrate that the Unfiltered and IG_mapped+Unmapped filtering methods, in addition to having the highest overall accuracy rates, are also the most flexible in terms of maintaining accuracy over differing sequencing parameters.

### Comparison of BALDR to alternate methods

A semi-*de novo* pipeline called BCR assembly from single cells (BASIC) has been recently developed for reconstructing Ig chains from single cells [37]. BASIC reconstructs the Ig sequence by anchoring reads to the V and the C genes and then extends the sequence by progressively stitching overlapping reads to the anchor sequence. We compared the performance of BASIC with BALDR on three B cell datasets and at varying sequencing parameters. When run using default values and hg19 reference, we obtained productive chains for 59% heavy (104/176) and 57% light (100/176) chains for the AW2-AW3 dataset using SE 151 base reads. The concordance of productive chains with RT-PCR-derived sequences based on the comparison of V(D)J genes and CDR3 sequence was 53% (61/115) for the heavy and 54% (76/140) for the light chains (Additional file 1: Table S6, Additional file 2). These accuracies were much lower than reported in the original study. As the dataset used in the BASIC study used 50 base reads, we trimmed our AW2-AW3 reads to 50 bases, retaining only the proximal ends of the read. Using the trimmed reads, the accuracy of reconstruction for productive chains was 93% for heavy and 97% for light chains (Additional file 1: Table S6). For the same trimmed reads, the IG_mapped+Unmapped method showed an accuracy of 98% for heavy and 99% for light chains. We also tested BASIC for the CD19+ Lin– B cell dataset which made use of paired-end 76-base reads. The accuracies for heavy and light chains were 93.5% and 100% for BASIC, while those for IG_mapped+Unmapped were 96.8% and 100%, respectively (Additional file 1: Table S6). Furthermore, we also compared the accuracy of BASIC in reconstructing Ig chains on a set of 86 plasmablasts under different conditions of read lengths and single-end or paired-end sequencing. We found that the accuracy of BASIC varies with the sequencing condition, ranging from 73.5% to 97% for IgH and from 95.1% to 97.6% for IgL. Overall, the accuracy of obtaining paired chains ranged from 70.8 to 91.7% for the different conditions. In contrast, the recommended IG_mapped+Unmapped method in the BALDR pipeline consistently shows high accuracies of 100% for IgH, 95.1–97.6% for IgL, and 95.8% for accurately obtaining paired IgH-IgL under all conditions. Overall, the IG_mapped+Unmapped method shows higher accuracy than BASIC, with significantly higher accuracy with longer reads, and maintains accuracy over a greater range of sequencing parameters.

### The BALDR pipeline accurately reconstructs Ig chains in rhesus macaques

**Fig. 7:**
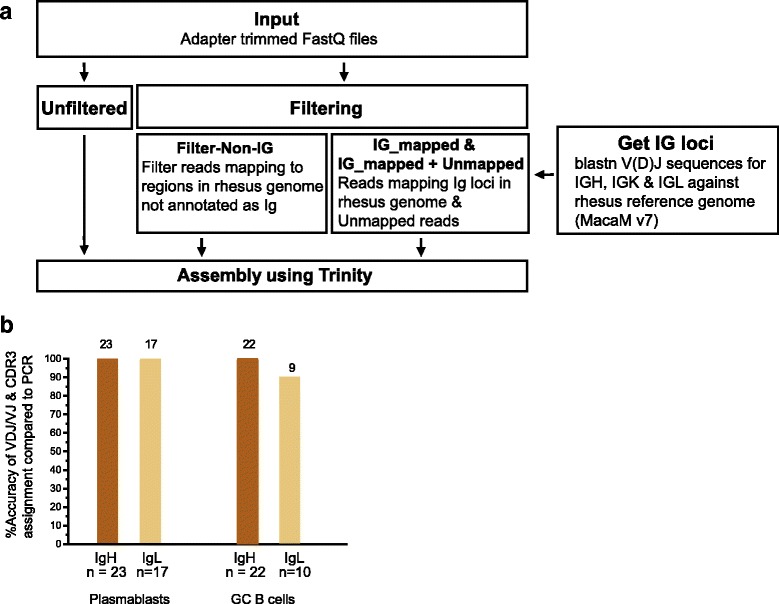
Ig transcript reconstruction in rhesus macaques with poor immunoglobulin reference annotation. **a** Pipeline for Ig assembly using unfiltered and filtered approaches (Filter-Non-IG: Discard reads mapping to non-Ig annotated regions of rhesus genome; IG_mapped: select reads mapped to the Ig coordinates and IG_mapped+Unmapped: combine IG_mapped reads and Unmapped reads for assembly). Ig reconstruction was carried out for 42 plasmablasts, 33 memory B cells, and 33 germinal center (GC) B cells. **b** Concordance of V(D)J gene annotation and CDR3 nucleotide sequence of Filter-Non-IG method with nested RT-PCR sequences from plasmablast and GC B cells

The rhesus macaque model is critical to the development of an AIDS vaccine. Historically, the majority of vaccines that demonstrate efficacy and achieve licensure elicit high levels of antibodies capable of neutralizing infection by the pathogen. To date, development of an HIV vaccine capable of generating neutralizing antibodies has remained elusive due to the high level of diversity in circulating viral strains. Nevertheless, several of the most promising HIV vaccine candidates have been capable of eliciting antibodies that exhibit moderate levels of neutralizing antibodies [38]. Despite its inherently high research value, the Ig loci in the rhesus macaque remain poorly annotated. There are currently 224 V(D)J genes for the rhesus macaque in the IMGT database [3]; however, it has been estimated that as many as 50% or more of Ig gene segments may be missing [39]. To enable reconstruction of antibody sequences in rhesus macaques, we designed and tested three Ig transcript filtering transcript strategies, taking into account the current state of rhesus macaque genome references (Fig. 7). Similar to the strategy for humans, we tested filtering strategies in which reads mapping to the immunoglobulin loci (IG_mapped), or to the Ig loci and also to reads that did not map to annotated, non-Ig genes (IG_mapped+Unmapped) were retained for reconstruction. In order to determine the Ig loci in the macaque MacaM v7 reference genome, rhesus V, D, J, and constant region sequences from the IMGT database, and those reported by Sundling [32] and more recently by Ramesh [33] were aligned to the genome fasta files using blastn. Once defined, these loci (details in Methods) were then used for mapping to identify and retain reads containing immunoglobulin sequences in our single-cell data. We also tested another strategy (Filter-Non-IG) where we aligned reads to the MacaM (v7) reference genome, all reads mapping to an annotated, non-immunoglobulin gene were discarded, and the remaining reads were retained for assembly. For annotation, we used the sequences available from IgBLAST (original source [32]).

We sequenced 42 plasmablasts, 33 splenic germinal center (GC) B cells, and 33 memory B cells, the latter of which were purified based on their specificity for epitopes in the experimental vaccine. For the rhesus plasmablast dataset, 42/42 cells had both IgH and IgL genes for which annotation was available; for the rhesus splenic B cells high confidence annotations could be made for 24 cells for both IgH and IgL. A productive chain was reconstructed for all plasmablasts with each method (Additional file 1: Figure S6A and Table S7, Additional file 2). The reconstruction success was 84.8% for IgH and IgL for the GC B cells and 81.8% for IgH and 100% for IgL for antigen-specific memory B cells using the Unfiltered method (Additional file 1: Table S7, Additional file 2). The Filter-Non-IG and the IG_mapped+Unmapped methods showed similar results, with Filter-Non-IG performing slightly better in the memory B cells. Lastly, the lowest number of productive reconstructions was obtained with the IG_mapped method (Additional file 1: Figure S6A and Table S7).

In order to determine the accuracy of reconstructions, we obtained the PCR sequence for the single cells. We were able to obtain high-quality PCR sequences for 23 IgH and 17 IgL from plasmablasts and 22 IgH and 10 IgL from GC B cells. Unfiltered, Filter-Non-IG, and IG_mapped+Unmapped showed the same high accuracy of 100% for IgH and IgL in plasmablasts and 100% for IgH and 90% for IgL (9/10) in GC B cells (Fig. 7b, Additional file 1: Figure S6B and Table S7). The discordant reconstruction differed only in the J gene assignment with the PCR (Additional file 2). The IG_mapped method showed high accuracies with plasmablast but showed very low accuracy for IgH (40.9%) in GC B cells.

We also assessed the computational time for assembly of each filtering method. The median time for assembly using the Unfiltered method was 19,701 s (328 min), 8020 s (134 min), and 5863 s (98 min) for memory B cells, GC B cells, and plasmablasts, respectively (Additional file 1: Figure S6C). The Filter-Non-IG method is two to three times faster than the Unfiltered method, while IG_mapped+Unmapped is 4–30 times faster than the Unfiltered method. Collectively, these data demonstrate that the BALDR pipeline can accurately reconstruct paired immunoglobulin genes from sc-RNA-seq data generated from rhesus macaque B cells.

## Discussion

In this study we report the utility of *de novo* assembly for the accurate reconstruction of the BCR heavy and light chain sequences from full-length single-cell RNA-seq data. We further tested the impact of various filtering methods and sequencing parameters on V(D)J sequence accuracy and recovery efficacy. Lastly, we present the optimal parameters for BCR reconstruction with a bioinformatics pipeline we refer to as BALDR (**B**CR **A**ssignment of **L**ineage using ***D****e novo* **R**econstruction). It is important to note that we have developed and validated the BALDR methodology using primary human B cells, namely vaccine-induced plasmablasts, and primary peripheral blood CD19+ B cells. Further, we have demonstrated that BALDR accurately reconstructs paired IgH + IgL sequences from B cells from rhesus macaques.

The ability to efficiently extract paired antigen receptor information from primary human immune cells *ex vivo* and link it with single-cell transcriptome data opens the way for powerful new analyses with clinical samples that were previously only possible in murine models. One attractive application of this technology is to perform “lineage-tracing” studies that link the transcriptional data from individual B cell clonotypes at specified differentiation states and then follow the “fates” of individual clones by repertoire sequencing. The clonotype sequence provided by the BALDR pipeline also makes it possible to generate monoclonal antibodies and thus link transcriptional information with functional qualities (e.g., affinity, neutralization activity) of the antibody. Here, we have used BALDR to extract IgH + IgL clonotypic information in vaccine-induced B cells; this clonotype sequence information can be used to monitor vaccine recipients over time and identify individual B cell lineages capable of differentiating into long-lived antibody-secreting plasma cells or persistent memory B cells and link it to transcriptional information. An alternative use of this tool is to link transcriptional state with clonotype-specific properties of the antibody, such as the proclivity to undergo class switching, SHM, or post-translational modifications. Used in this way, the application of BALDR and sc-RNA-seq to primary B cells induced in human vaccination studies also provides a novel analytic tool to the emerging field of “systems vaccinology” in which high-throughput technologies are used to identify factors predicting vaccine efficacy [40].

We evaluated different filtering strategies and found that the most accurate strategy was to retain reads that (1) mapped to the three defined immunoglobulin loci in the GRCh38 genome and (2) did not map to an annotated gene. This method, IG_mapped+Unmapped, identified the correct clonotype in 99.2% (253/255) of paired chains and correctly paired IgH + IgL information in 96.9% (93/96) cells. The accuracy of our pipeline compares favorably with recent reports using similar approaches for T cells where the accuracies ranged from 77.5% (14/20 α chain and 17/20 β chain) [17] to 78.4% [16]. In both the human and rhesus datasets, the inclusion of unmapped reads for Ig reconstruction improved the recovery rate and accuracy rate of the reconstructed chains compared to strategies that relied on inclusion of reads mapping to a reference. This advantage becomes increasingly important when analyzing human populations or models with poor representation of alleles in IMGT, or as we demonstrated, for B cell populations with high levels of SHM. Indeed, inclusion of the unmapped reads also provides more flexibility with respect to the read length used as input data, since shorter reads may not map to highly variable regions of Ig chains during the pre-filtering stage. The IG_mapped+Unmapped method involves mapping the reads to the reference genome with STAR, which allows us to simultaneously obtain the transcript quantification needed for pairing of the transcriptome information. For the rhesus, where the Ig loci are not well annotated in the genome, using this strategy of the Filter-Non-IG method provides nearly identical results to using all reads (Unfiltered method), at the same time reducing the computation time to almost half.

We have not looked specifically at the effect of sequencing depth on the Ig reconstruction. However, our datasets ranged from ~ 400,000 reads to 4 million reads, and we were able to get a high rate of reconstruction in most samples. For analyzing the transcriptome, a sequencing depth of 1 million reads per cell has been recommended for saturated gene detection [41] in sc-RNA-seq. When analyzing plasmablasts, where 5–50% of the mRNA transcripts can be immunoglobulins, a secondary consideration is achieving sufficient depth for the remaining transcriptional analysis, and we typically target for ~ 1.5 to 2 million reads per single plasmablast. For conventional B cells, we observed reads attributed to immunoglobulin to be less than 8%, and a sequencing depth of 1–1.5 million reads is adequate to capture the transcriptome along with Ig reconstruction.

All filtering methods described in the current study are made available in the BALDR pipeline. We recommend using IG_mapped+Unmapped for human cells and the Filter-Non-IG method for rhesus macaques. The transcript quantification that is obtained simultaneously with these methods can be used to carry out gene expression analysis. Further improvements in the pipeline will involve adapting the Unfiltered method towards organisms with low-quality/missing reference genomes. Additionally, improving the Ig annotations for rhesus will result in higher accuracy for the IG_mapped+Unmapped method while reducing the computation time significantly.

One of the key strengths of the BALDR pipeline is its ability to generate accurate Ig transcript reconstructions for samples in which genomic references of immunoglobulin gene sequences are lacking. We demonstrated this activity by reconstructing Ig transcripts from single B cells obtained from rhesus macaques after vaccination with experimental vaccines. Currently, resources for Ig annotation in the rhesus macaque are underdeveloped. For example, the IMGT database contains 19 immunoglobulin heavy chain variable (IGHV) genes, despite estimates that up to 60 genes are present in the rhesus immunoglobulin IgH loci [3, 39]. Efforts to improve genomic resources of the Indian rhesus macaque immunoglobulin loci are currently underway, and a high density map of the rhesus immunoglobulin loci has recently been published [33] and will be an important advance for AIDS vaccine development. However, it will be some time before the allelic diversity of the immunoglobulin genes is characterized for the North American captive rhesus macaque population. The BALDR pipeline maintains high accuracy of Ig transcript reconstruction when input data are from a species with scant annotation of the Ig loci, such as currently exist for the rhesus macaque, and thus confident analysis of sc-RNA-seq data can be applied to current ongoing studies in the macaque model.

The independence of the BALDR pipeline from high-quality Ig reference sequences may also have added utility for human vaccine studies, particularly in populations in Africa and Asia, where allelic diversity is relatively uncharacterized. In a recent study by Morris and colleagues, analysis of 28 HIV-infected women in South Africa characterized approximately 130 IGHV alleles that were not represented in the IMGT database [42]. In these scenarios, bioinformatic tools that rely on mapping to an Ig reference are likely to have higher rates of incorrect or abortive clonotype reconstructions. In these populations, the BALDR pipeline can be particularly useful for sc-RNA-seq studies of HIV-specific B cells or to enhance the recovery of paired IgH + IgL sequences and accelerate discovery of novel antibodies capable of neutralization breadth against HIV.

The BALDR pipeline requires sequence information across the entirety of the BCR variable region. This requirement necessitates that the NGS library be prepared separately for each cell, so that sequence fragments across the full length of transcripts can be barcoded. These whole-transcript methods (e.g., SMART-Seq) have been extensively used for sc-RNA-seq in the literature, but they have the drawback of being relatively expensive. Recently, several novel technologies for obtaining large numbers of single-cell transcriptomes at low cost have been reported including the use of nanowells (ICELL8) [43] and emulsion droplets (Drop-seq [44], inDrop [45], 10X Genomics [46]). These methods are able to drastically reduce the cost per transcriptome by incorporating cell barcodes during reverse transcription, eliminating the need for library preparation on each cell. One consequence to these approaches, however, is that only 3′ sequence information is retained and they are unable to capture sequence across the 5′ variable region of Ig transcripts. However, while SMART-Seq (as used in this study) and other well-based techniques are capable of generating high-quality transcriptome data with accurate clonotype information, the cost and low throughput are significant limitations. Ongoing improvements in automation and reduction in sequencing costs have mitigated these factors somewhat, and studies including > 5000 SMART-Seq transcriptomes have been published [47]. For most labs, however, datasets comprising a few hundred cells are practical, and are best suited for populations where the clonotypes of interest are enriched (e.g., antigen-specific cells), rather than for large-scale screening of paired repertoires.

One potential alternate use for the BALDR pipeline is for antibody cloning. Existing methodology uses primers specific for the V region followed by extensive PCR to obtain antibody sequences from plasmablasts [19, 48]. On a technical level, sc-RNA-seq combined with BALDR Ig reconstruction offers some advantages over traditional cloning. (1) The recovery of IgH + IgL sequences is highly efficient, at near 100% for plasmablasts and total B cells, and > 80% for antigen-specific memory B cells. Whereas this difference is marginal for reported cloning efficiencies for human plasmablasts (~ 70–80%) [19], it differs more significantly for non-plasmablast B cells with lower levels of immunoglobulin transcripts, and for plasma cells from rhesus macaques, where efficiencies are < 50% [22]. (2) Because BALDR has the ability to quantitate reconstructed Ig chains and select the most abundant chains, it is relatively resistant to interwell contamination. (3) Lastly, the use of template switching rather than multiplex priming at the 5′ end of the Ig transcript provides greater utility for recovery of antibodies in populations or animal models with poorly characterized V genes. Despite these advantages, sc-RNA-seq is about twice the cost per recovered Ig pair compared to conventional cloning, and it requires access to bioinformatics expertise; thus, the utility of BALDR for antibody cloning may be limited to unique circumstances (such as cloning from rhesus macaques). However, the continuing decline of sc-RNA-seq costs may lead to a more general use of sc-RNA-seq for antibody recovery.

## Conclusions

Here, we have developed and validated a novel bioinformatics pipeline capable of accurate reconstruction of antibody gene sequences in humans and other animal models from sc-RNA-seq data, which offers flexibility in the sequencing format requirements of input data. The BALDR pipeline allows linking of sc-RNA-seq transcriptome data of individual B cells with antibody clonotype information and will likely have broad utility for dissecting antibody responses in vaccine studies and for longitudinal “lineage-tracing” studies in which clonotype data tracked over time can be mapped back to early B cell transcriptome information.

To enable open access to our method by researchers analyzing B cells using sc-RNA-seq, we have made all necessary scripts and supporting documentation to run the BALDR tool freely available for download (https://github.com/BosingerLab/BALDR). Additionally, to enable further advancement and refinement of bioinformatic strategies to reconstruct antibody genes, we have made available the validation dataset containing paired NGS + Sanger sequence data. The ability to link clonal dynamics, antibody specificity, and transcriptional information of antigen-specific B cells is likely to be of widespread use for multiple fields of immunology and genomics and to provide novel molecular insight into multiple aspects of B lymphocyte biology.

## Additional files

Additional file 1; Supplementary figures and tables. (PDF 8130 kb)

Additional file 2: Results of Ig reconstruction using BALDR. The V(D)J gene annotations, CDR3 sequences, the number of reads mapping to the Ig chain using bowtie2, whether the chain is productive, and the complete sequences are shown for the Ig chains reconstructed using BALDR pipeline for all the human datasets (AW2-AW3 (SE151) plasmablast dataset with and without *in silico* read normalization, plasmablast AW1 (PE101, PE75, PE50, SE101, SE75, and SE50), the VH (PE76) CD19+ Lin– B cell dataset, and the AW2-AW3 (SE50) for IG_mapped+Unmapped method) and the rhesus macaque datasets (BL8, BL6.1, and BL6.2). When the RT-PCR sequence is available, the V(D)J genes and the CDR3 sequence are also shown for the corresponding chains, and concordance between the BALDR reconstructed chains and the RT-PCR sequence is indicated. The results for Ig reconstruction using the BASIC method are also shown along with matching RT-PCR for AW2-AW3 (SE101 and SE50), VH (PE76), and AW1 (PE101, PE75, PE50, SE101, SE75, and SE50) datasets. (XLSX 2190 kb)

Additional file 3: Clonal assignments for human single-cell datasets. The single cells were assigned to clonal families based on the V, J and CDR length for paired IGH and IgL chains. (XLSX 46 kb)

Additional file 4: Discordant reconstructions for AW2_AW3 dataset IgH chains. The V, D, J genes, CDR3 sequences, and complete reconstructed sequence are shown for discordant IgH reconstructions along with annotations for Ig reconstruction with Unfiltered methods and the PCR sequence. Also included are models that were filtered in the BALDR pipeline, as they were not predicted to be productive. (XLSX 18 kb)

Additional file 5: Somatic hypermutations in human single-cell datasets. The number of somatic hypermutations for AW2_AW3 plasmablast and VH CD19+ Lin– single cells compared to the IMGT germline sequences. (XLSX 19 kb)

Additional file 6: Percentage of immunoglobulin reads in human plasmablasts and CD19+ Lin– B cells. The percentage of Ig reads is calculated by dividing the number of reads mapping to the top model to the total number of reads for AW2-AW3 plasmablast dataset and VH CD19+ Lin– B cell dataset. (XLSX 23 kb)

Additional file 7: Sequences from nested RT-PCR. The Ig chains obtained from Sanger sequencing of nested RT-PCR. (XLSX 62 kb)

## Abbreviations

BALDR: BCR Assignment of Lineage by De novo Reconstruction
D: Diversity gene segments
HIV: Human immunodeficiency virus
Ig: Immunoglobulin(s)
IGH: Immunoglobulin heavy chain
IgH: Immunoglobulin heavy chain
IGK: Immunoglobulin kappa light chain
IGL: Immunoglobulin lambda light chain
IgL: Immunoglobulin light chain
J: Joining gene segments
NGS: Next generation sequencing
PBMC: Peripheral blood mononuclear cell
RT-PCR: Reverse transcription polymerase chain reaction
sc-RNA-seq: Single-cell RNA-seq
SIV: Simian immunodeficiency virus
TCR: T-cell receptor
V: Variable gene segments
