# Supplementary material for: BALDR: a computational pipeline for paired heavy and light chain immunoglobulin reconstruction in single-cell RNA-seq data
Source: Genome Med. 2018 Mar 20;10:20. doi: 10.1186/s13073-018-0528-3 (PMC5859752; doi:10.1186/s13073-018-0528-3)
Supplement: Supplementary file 1 — Supplementary figures and tables. (PDF 8130 kb) [file 13073_2018_528_MOESM1_ESM.pdf]

Supplementary Information

Supplementary Figures

Figure S1. The number of reads mapped to the IgH and IgL models for 176 human plasmablasts (AW2-AW3 dataset) using bowtie2 reconstructed from (A) Unfiltered (B) IG_mapped (C) IMGT_mapped and (D) Recombinome_mapped methods. Also shown are the ratios of the number of reads mapped for the top two IgH and IgL models for each method. The dashed line indicates a two-fold ratio. The median is shown as a red line.

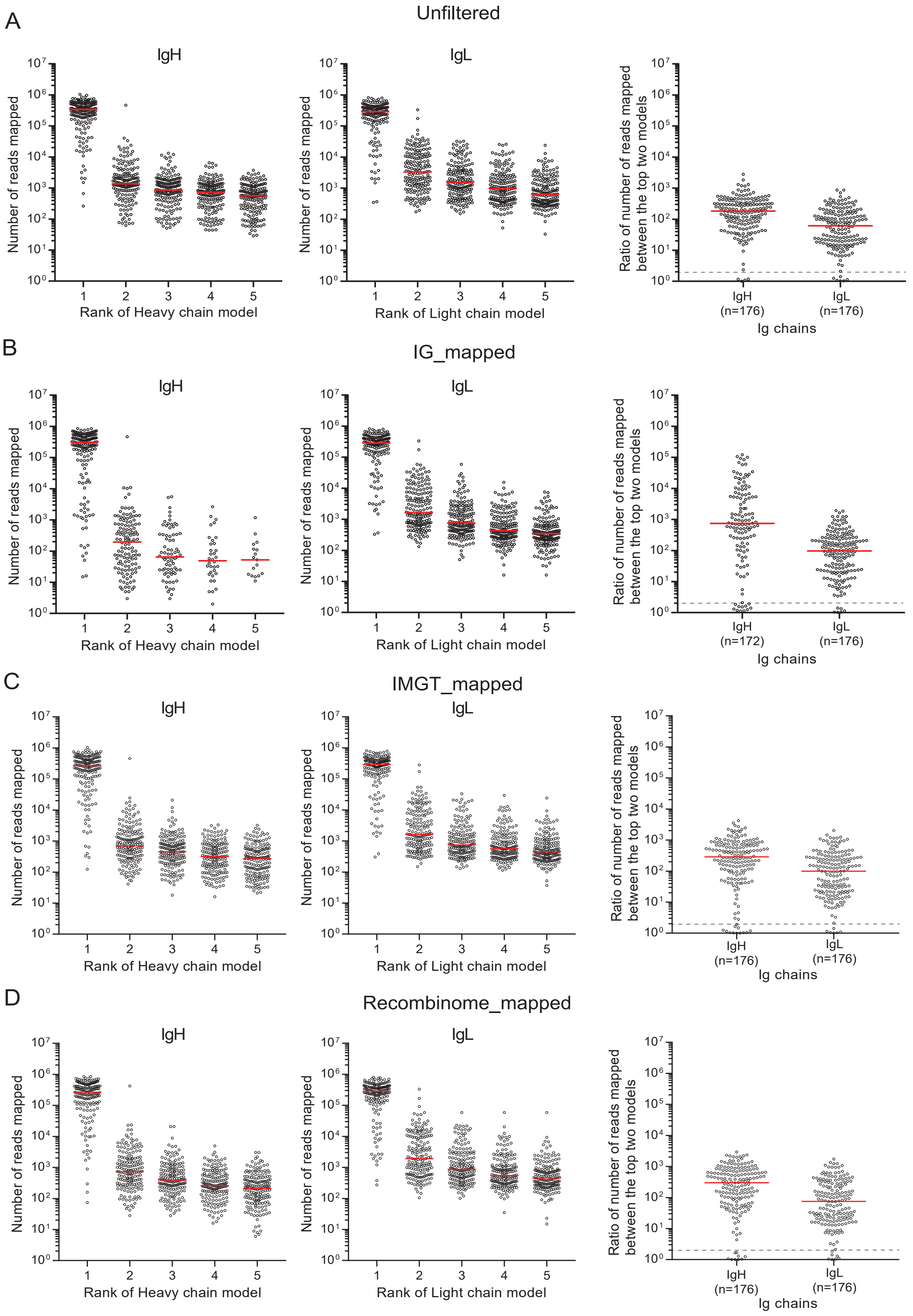
A
Unfiltered
IgH
IgL
Number of reads mapped
Rank of Heavy chain model
Rank of Light chain model
Ratio of number of reads mapped between the top two models
IgH (n=176)
IgL (n=176)
Ig chains
B
IG_mapped
IgH
IgL
Number of reads mapped
Rank of Heavy chain model
Rank of Light chain model
Ratio of number of reads mapped between the top two models
IgH (n=172)
IgL (n=176)
Ig chains
C
IMGT_mapped
IgH
IgL
Number of reads mapped
Rank of Heavy chain model
Rank of Light chain model
Ratio of number of reads mapped between the top two models
IgH (n=176)
IgL (n=176)
Ig chains
D
Recombinome_mapped
IgH
IgL
Number of reads mapped
Rank of Heavy chain model
Rank of Light chain model
Ratio of number of reads mapped between the top two models
IgH (n=176)
IgL (n=176)
Ig chains

Figure S1

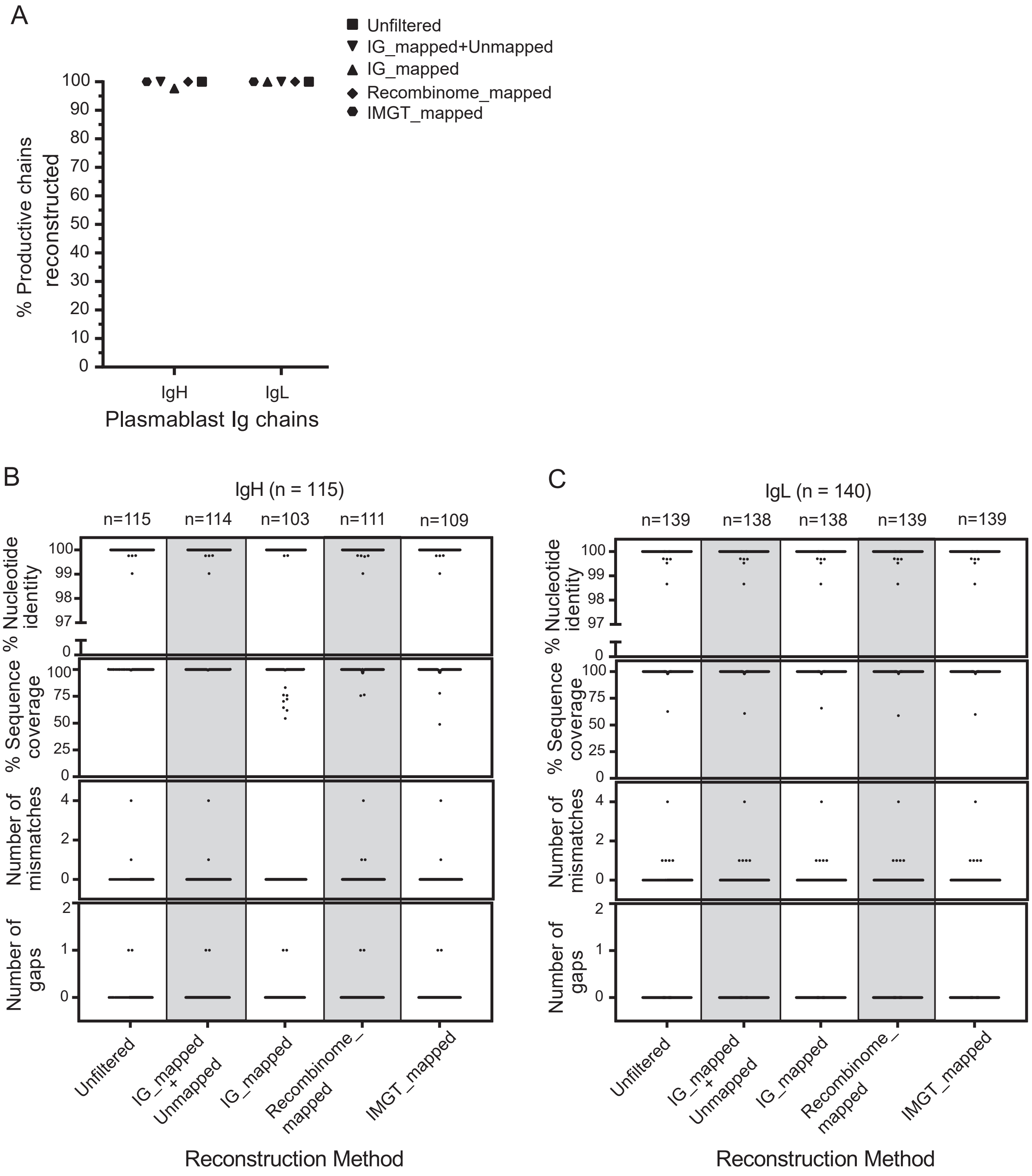

Figure S2. Recovery and accuracy of BALDR reconstruction. (A) The recovery rate of productive Ig chains by bioinformatic reconstruction shown as a percentage of all analyzed cells (176 plasmablasts) for IgH (heavy) and IgL (light) chains reconstructed from (AW2-AW3 dataset). (B) Comparison of reconstructed IgH transcripts with PCR nucleotide sequence by blastn (top high scoring segment pair was selected). The number of reconstructed IgH chains used for nt alignment for each method is indicated on the top. Sequence coverage calculated as: alignment length x 100 / length of PCR sequence. (C) blastn results for alignment of accurately reconstructed IgL to PCR. Black horizontal lines in (B) and (C) indicate conglomerate of data points at 100% or 0.

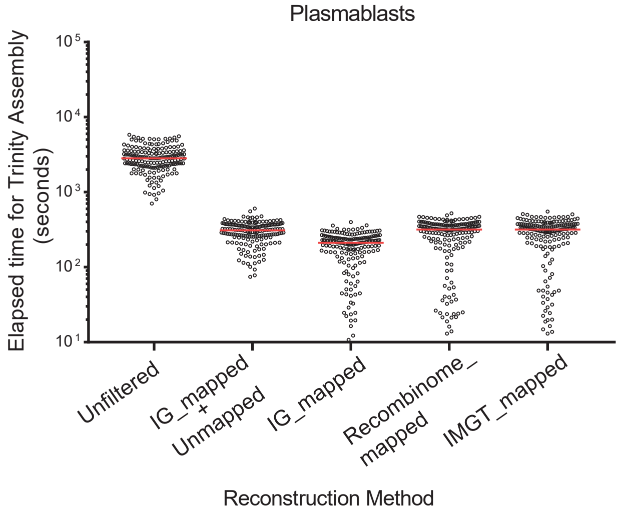

Figure S3. Elapsed time in seconds for Trinity assembly of 176 plasmablasts (AW2-AW3 dataset). The assemblies were run by executing 8 jobs simultaneously, each with 8 threads and 32GB RAM on Amazon Web Services EC2 m4.16xlarge instances (Intel Xeon E5-2676 v3, 64 cores and 256GB RAM. The median is shown as a red line. The time reported by the bash time command is shown.

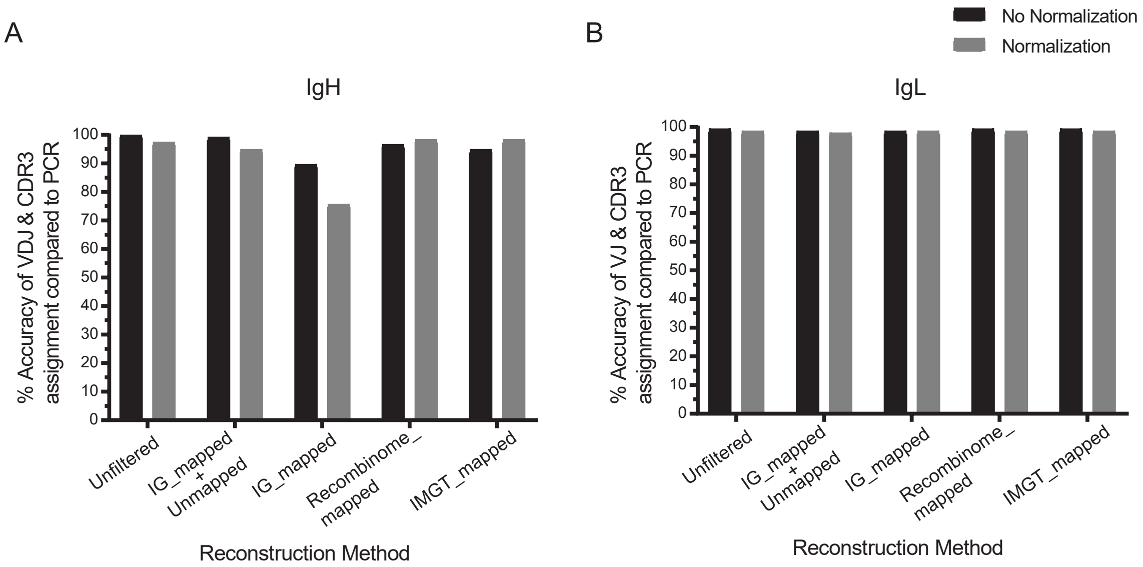

Figure S4. Effect of in silico read normalization on Ig reconstruction accuracy in human plasmablasts (AW2-AW3 dataset). Trinity assembly was carried out with and without normalization. Accuracy of Ig reconstruction of the IgH (A) and IgL (B) was determined by comparison to the 115 IgH and 140 IgL sequences obtained from nested RT-PCR and Sanger sequencing.

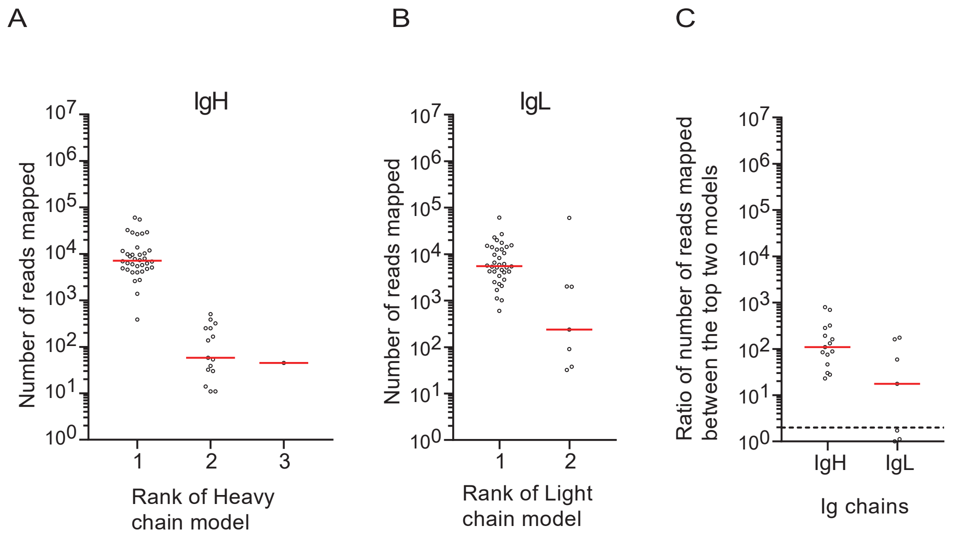

Figure S5. A single dominant transcript model for IgH and IgL also obtained in conventional human CD19+ B cells The number of reads mapped to the reconstructed models using bowtie2 for IgH (A) and IgL (B) chains and the ratio of top two models for IgH and IgL (C). The dashed line indicates a two-fold ratio. The median is shown as a red line.

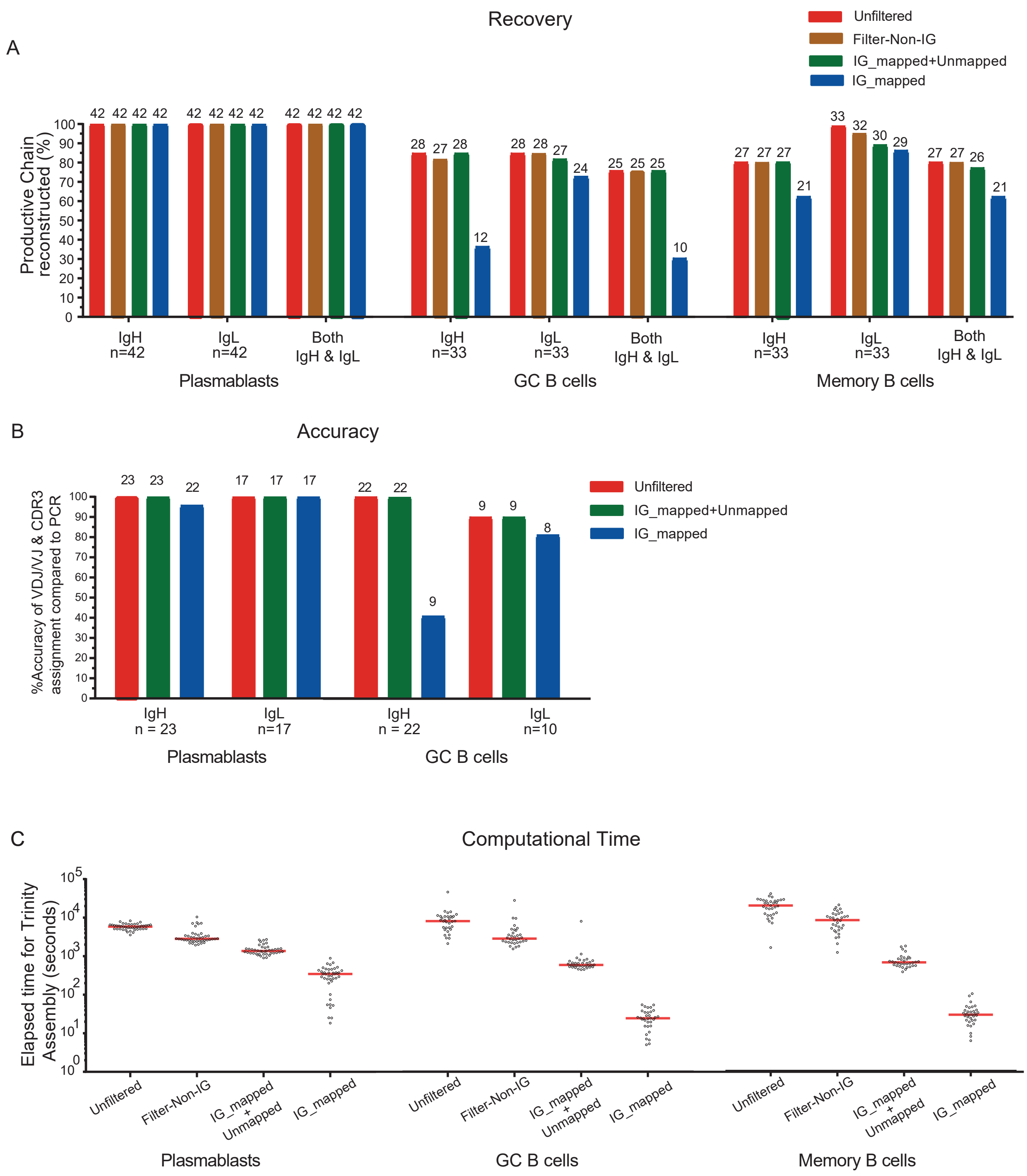

Figure S6. Recovery and accuracy of Ig transcript reconstruction in rhesus macaques. (A) The percentage of productive chains reconstructed using the four different methods. (B) Concordance of V(D)J gene annotation and CDR3 nucleotide sequence of Ig transcripts obtained from Unfiltered, IG_mapped+Unmapped and IG_mapped methods with nested RT-PCR sequences for plasmablasts and GC B cells. (C) Elapsed time in seconds for Trinity assembly. The assemblies were run by executing 4 jobs simultaneously, each with 8 threads and 32 GB RAM on a local Dell PowerEdge R630 Server (Intel Xeon E5-2620 v4, 16 cores/32 threads, 196 GB RAM). The time reported by the bash time command is shown.

Supplementary Tables

Supplementary Table S1. Datasets used in the study.

Dataset	Species	Cell Type	Num of cells	SE/ PE	Read Length	Average Depth (million)	Sequencer	Nested RT-PCR		
								Heavy	Light	Both heavy & light from the same cell
AW1*	Human	Plasmablasts	86	PE	101	2.86 (0.6-4.9)	Illumina HiSeq 3000	34	41	24
AW2-AW3	Human	Plasmablasts	176	SE	151	1.63 (0.4-2.3)	Illumina HiSeq 3000	115	140	96
VH**	Human	CD19+	36	PE	76	0.7 (0.4-1.9)	Illumina MiSeq	31[#]	31[#]	26
BL6.1*	Rhesus	Germinal Center	33	SE	101	3.57 (2.8-4.4)	Illumina HiSeq 3000	22	10	8
BL6.2*	Rhesus	Memory	33	SE	101	3.48 (1.5-4.4)	Illumina HiSeq 3000	NA	NA	NA
BL8	Rhesus	Plasmablasts	42	PE	101	2.19 (1.4-2.8)	Illumina HiSeq 3000	23	17	8

*Datasets sequenced twice to get greater depth. The average depth reported is after combining the two runs

**VH dataset – 30 single cells were sequenced by a modified SMARTer protocol while the 6 single cells were sequenced by the conventional SMARTer protocol (see Methods)

[#] The nested RT-PCR sequences were obtained from the amplified SMART-Seq v4 library

Supplementary Table S2. Accuracy of Ig reconstruction for AW2-AW3 human plasmablast dataset

**Method**	**Productive chains**		**Concordance of V(D)J annotation & CDR3 sequence with nested RT-PCR sequences**		
	**Heavy (n=176)**	**Light (n=176)**	**Heavy (n=115)**	**Light (n=140)**	**Both heavy & light from the same cell (n=96)**
Unfiltered	176 (100%)	176 (100%)	115 (100%)	139 (99.3%)	95 (98.9%)
IG_mapped+Unmapped	176 (100%)	176 (100%)	114 (99.1%)	139 (99.3%)	94 (97.9%)
Recombinome_mapped	176 (100%)	176 (100%)	111 (96.5%)	139 (99.3%)	91 (94.8%)
IMGT_mapped	176 (100%)	176 (100%)	109 (94.8%)	139 (99.3%)	89 (92.7%)
IG_mapped	172 (97.7%)	176 (100%)	103 (89.6%)	139 (99.3%)	85 (88.5%)

Supplementary Table S3. Accuracy of Ig reconstruction for AW2-AW3 human plasmablast dataset considering clonotypes.

Method	Concordance of V(D)J annotation & CDR3 sequence with nested RT-PCR sequences[$]								
	Heavy (n=67)			Light (n=80)			Both heavy & light from the same cell (n=58)		
	Yes	No	Yes/ No*	Yes	No	Yes/ No*	Yes	No	Yes/ No*
Unfiltered	67 (100%)	0	0	79 (98.8%)	1	0	57 (98.3%)	1	0
IG_mapped +Unmapped	66 (98.5%)	1	0	79 (98.8%)	1	0	56 (96.6%)	2	0
Recombinome_ mapped	64 (95.5%)	1	2	79 (98.8%)	1	0	54 (93.1%)	2	2
IMGT_mapped	63 (94%)	2	2	79 (98.8%)	1	0	53 (91.4%)	3	2
IG_mapped	58 (86.6%)	6	3	79 (98.8%)	1	0	49 (84.5%)	6	3

[$]The cells are collapsed into clonal families and the accuracy is determined for each family.

[*]Cases where all members in a clonal family did not have the same V(D)J and CDR3 as the corresponding PCR sequences

Supplementary Table S4. Accuracy of Ig reconstruction for CD19+Lin- B cells (VH dataset).

**Method**	**Productive chains**		**Concordance of V(D)J annotation & CDR3 sequence with nested RT-PCR sequences**		
	**Heavy (n=36)**	**Light (n=36)**	**Heavy (n=31)**	**Light (n=31)**	**Both heavy & light from the same cell (n=26)**
Unfiltered	36 (100%)	36 (100%)	30 (96.8%)	31 (100%)	25 (96.2%)
IG_mapped+Unmapped	36 (100%)	36 (100%)	30 (96.8%)	31 (100%)	25 (96.2)
Recombinome_mapped	35 (97.2%)	36 (100%)	29 (93.5%)	31 (100%)	24 (92.3)
IMGT_mapped	34 (94.4%)	36 (100%)	28 (90.3%)	31 (100%)	24 (92.3)
IG_mapped	34 (94.4%)	36 (100%)	28 (90.3%)	31 (100%)	24 (92.3)

Supplementary Table S5. Accuracy of Ig reconstruction for the BALDR pipeline (IG_mapped+Unmapped) compared to the BASIC method

Dataset	Cell Type	Read length	Concordance of V(D)J annotation & CDR3 sequence with nested RT-PCR sequences					
			Heavy chain		Light chain		Both heavy & light from the same cell	
			BASIC	BALDR	BASIC	BALDR	BASIC	BALDR
AW2-AW3	Plasmablast CD27hiCD38hi	SE 151	61/115 (53%)	114/115 (99.1%)	76/140 (54.3%)	138/140 (98.6%)	55/96 (57.3%)	94/96 (97.9%)
AW2-AW3	Plasmablast CD27hiCD38hi	SE 50	107/115 (93%)	113/115 (98.3%)	136/140 (97.1%)	139/140 (99.3%)	87/96 (90.6%)	93/96 (96.8%)
VH	$CD19^{+}$Lin-	PE 76	29/31 (93.5%)	30/31 (96.8%)	31/31 (100%)	31/31 (100%)	24/26 (92.3)	25/26 (96.2%)
AW1	Plasmablast CD27hiCD38hi	SE 101	33/34 (97%)	34/34 (100%)	40/41 (97.6%)	40/41 (97.6%)	22/24 (91.7%)	23/24 (95.8%)
AW1	Plasmablast CD27hiCD38hi	SE 75	29/34 (85.3%)	34/34 (100%)	40/41 (97.6%)	40/41 (97.6%)	21/24 (87.5%)	23/24 (95.8%)
AW1	Plasmablast CD27hiCD38hi	SE 50	28/34 (82.4%)	34/34 (100%)	39/41 (95.1%)	40/41 (97.6%)	18/24 (75%)	23/24 (95.8%)
AW1	Plasmablast CD27hiCD38hi	PE 101	29/34 (85.3%)	34/34 (100%)	39/41 (95.1%)	40/41 (97.6%)	19/24 (79.2%)	23/24 (95.8%)
AW1	Plasmablast CD27hiCD38hi	PE 75	26/34 (76.5%)	34/34 (100%)	39/41 (95.1%)	39/41 (95.1%)	17/24 (70.8%)	23/24 (95.8%)
AW1	Plasmablast CD27hiCD38hi	PE 50	25/34 (73.5%)	34/34 (100%)	40/41 (97.6%)	40/41 (97.6%)	17/24 (70.8%)	23/24 (95.8%)

The comparison between BALDR and BASIC was carried out for the AW2_AW3 (176 cells; PCR available: 115 IgH and 140 IgL), VH (36 cells; PCR available: 31 IgH and 31 IgL) and AW1(86 cells; PCR available: 34 IgH and 41 IgL). The number of productive reconstructed Ig chains having the same V(D)J & CDR3 assignment as the corresponding PCR sequence are indicated out of the total number of available PCR sequences.

Supplementary Table S6. Accuracy of Ig reconstruction for AW1 human plasmablast datasets.

**Chain**	**Method**	**Paired end**			**Single end**		
		**101**	**75**	**50**	**101**	**75**	**50**
Heavy (n=34)	Unfiltered	34 (100%)	34 (100%)	34 (100%)	34 (100%)	34 (100%)	34 (100%)
	IG_mapped+Unmapped	34 (100%)	34 (100%)	34 (100%)	34 (100%)	34 (100%)	34 (100%)
	Recombinome_mapped	31 (91.2%)	32 (94.1%)	24 (70.6%)	33 (97.1%)	30 (88.2%)	1 (2.9%)
	IMGT_mapped	33 (97.1%)	33 (97.1%)	10 (29.4%)	33 (97.1%)	33 (97.1%)	11 (32.4%)
	IG_mapped	34 (100%)	33 (97.1%)	18 (52.9%)	21 (61.8%)	5 (14.7%)	0 (0%)
Light (n=41)	Unfiltered	40 (97.6%)	40 (97.6%)	40 (97.6%)	40 (97.6%)	40 (97.6%)	40 (97.6%)
	IG_mapped+Unmapped	40 (97.6%)	39 (95.1%)	40 (97.6%)	40 (97.6%)	40 (97.6%)	40 (97.6%)
	Recombinome_mapped	40 (97.6%)	40 (97.6%)	40 (97.6%)	40 (97.6%)	38 (92.7%)	35 (85.4%)
	IMGT_mapped	40 (97.6%)	40 (97.6%)	32 (78%)	40 (97.6%)	40 (97.6%)	38 (92.7%)
	IG_mapped	39 (95.1%)	40 (97.6%)	40 (97.6%)	39 (95.1%)	37 (90.2%)	22 (53.7%)
Both heavy & light from the same cell (n=24)	Unfiltered	23 (95.8%)	23 (95.8%)	23 (95.8%)	23 (95.8%)	23 (95.8%)	23 (95.8%)
	IG_mapped+Unmapped	23 (95.8%)	23 (95.8%)	23 (95.8%)	23 (95.8%)	23 (95.8%)	23 (95.8%)
	Recombinome_mapped	20 (83.3%)	23 (95.8%)	22 (91.7%)	22 (91.7%)	21 (87.5%)	5 (20.8%)
	IMGT_mapped	22 (91.7%)	22 (91.7%)	5 (20.8%)	22 (91.7%)	22 (91.7%)	7 (29.2%)
	IG_mapped	23 (95.8%)	22 (91.7%)	11 (45.8%)	15 (62.5%)	3 (12.5%)	0 (0%)

Supplementary Table S7. Recovery and Accuracy of Ig reconstruction for rhesus macaque single cell datasets.

**Cell type**	**Method**	**Number of productive chains reconstructed**			**Concordance of V(D)J annotation & CDR3 sequence with nested RT-PCR sequences**		
		**Heavy**	**Light**	**Both heavy and light**	**Heavy**	**Light**	**Both heavy and light**
Plasmablast (n=42)	Unfiltered	42/42 (100%)	42/42 (100%)	42/42 (100%)	23/23 (100%)	17/17 (100%)	8/8 (100%)
	Filter-Non-IG	42/42 (100%)	42/42 (100%)	42/42 (100%)	23/23 (100%)	17/17 (100%)	8/8 (100%)
	IG_mapped +Unmapped	42/42 (100%)	42/42 (100%)	42/42 (100%)	23/23 (100%)	17/17 (100%)	8/8 (100%)
	IG_mapped	42/42 (100%)	42/42 (100%)	42/42 (100%)	22/23 (95.7%)	17/17 (100%)	8/8 (100%)
GC B cells (n=33)	Unfiltered	28/33 (84.8%)	28/33 (84.8%)	25/33 (75.7%)	22/22 (100%)	9/10 (90%)	7/8 (87.5%)
	Filter-Non-IG	27/33 (81.8%)	28/33 (84.8%)	25/33 (75.7%)	22/22 (100%)	9/10 (90%)	7/8 (87.5%)
	IG_mapped +Unmapped	28/33 (84.8%)	27/33 (81.8%)	25/33 (75.7%)	22/22 (100%)	9/10 (90%)	7/8 (87.5%)
	IG_mapped	12/33 (36.4%)	24/33 (72.7)	10/33 (30.3)	9/22 (40.9%)	8/10 (80%)	3/8 (37.5%)
Memory B cells (n=33)	Unfiltered	27/33 (81.8%)	33/33 (100%)	27/33 (81.8%)	NA	NA	NA
	Filter-Non-IG	27/33 (81.8%)	32/33 (97%)	27/33 (81.8%)	NA	NA	NA
	IG_mapped +Unmapped	27/33 (81.8%)	30/33 (90.9%)	26/33 (78.8%)	NA	NA	NA
	IG_mapped	21/33 (63.6%)	29/33 (87.9%)	21/33 (63.6%)	NA	NA	NA
